# Low‐contact and high‐interconnectivity pathology (LC&HI Path): post‐COVID19‐pandemic practice of pathology

**DOI:** 10.1111/his.14174

**Published:** 2020-07-28

**Authors:** Mark J Arends, Manuel Salto‐Tellez

**Affiliations:** ^1^ Division of Cancer University of Edinburgh Cancer Research UK Edinburgh Centre IGMM Western General Hospital Campus Edinburgh UK; ^2^ Precision Medicine Centre of Excellence Centre for Cancer Research and Cell Biology Queen's University Belfast Belfast UK; ^3^ Cellular Pathology Belfast Health and Social Care Trust Belfast UK

**Keywords:** COVID‐19, digital pathology, pathology, telepathology

## Abstract

The COVID‐19 pandemic situation may be viewed as an opportunity to accelerate some of the ongoing transformations in modern pathology. This refers primarily to the digitalisation of the practice of tissue and cellular pathology diagnostics. However, it is also an opportunity to analyse the *modus operandi* of a discipline that has been practised in a similar manner for more than 100 years. The challenge is to define the next generation of interconnectivity tools that would be necessary to achieve a new operational model that, while ensuring low face‐to‐face interaction between the main players of the diagnostic pipeline, allows maximum interconnectivity to serve our patients and the immediate teaching and research needs associated with clinical tissue/cellular samples. This viewpoint aims to describe what this new paradigm, a low‐contact and high‐interconnectivity pathology (LC&HC Path) operation, may require in the near future.

## Introduction

At the moment in which this manuscript sees the light (mid‐2020), the world is in the midst of an unprecedented SARS‐CoV‐2/COVID‐19 pandemic of unknown consequences. With millions of people infected and hundreds of thousands of hospital deaths on test‐positive patients worldwide, the COVID‐19 pandemic is transforming the way we deliver virology, respiratory medicine and critical care; however, its influence is percolating into the way we practise medicine as a whole. Recently, both the Royal College of Pathologists and the Association for Pathology Informatics have called for a ‘relaxation’ of the regulatory mechanisms for the validation and implementation of digital pathology in routine diagnostics in these challenging times.^1^, ^2^ These guidelines were originally proposed by the Royal College and the Clinical Laboratory Improvement Amendments of 1988 (CLIA), respectively. In particular, it is considered that^1^:

- Pathologists who have limited or no validation, or who have not used digital pathology before, will find that they can confidently report some or many cases digitally, without undertaking a formal 1–2‐month validation comparing glass slides and digital images, but should be aware of the risks and mitigate these risks where possible.
- In exceptional circumstances they may decide to report cases digitally, using a risk mitigation approach – this does not remove the need for validation or quality assurance once normal services are being provided.

While a definitive analysis of its effects on tissue pathology diagnostics will require the benefit of time and perspective, we feel it is important to highlight the needs we perceive currently, as practising pathologists, while we are still adapting old structures to a new reality as the pandemic is still active, so that these can be taken into account in the near future.

## Hierarchy of connectivity tools

**Figure 1:**
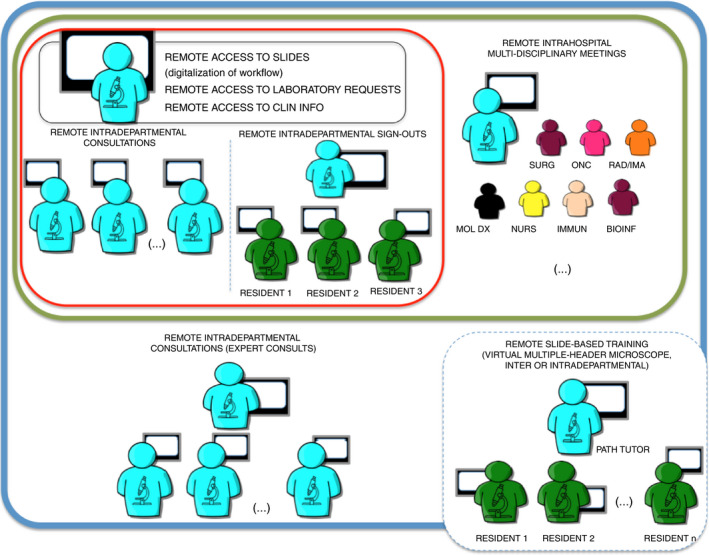
Depiction of levels of interconnectivity required to facilitate a satisfactory remote pathology operation. The black frame includes requirements for an individual diagnostic operation; the red frame shows intradepartmental activities, both in diagnostic consultation and sign‐outs; the green frame depicts the connectivity required for a successful intrahospital clinical consultation system, the most complex version of which is the multidisciplinary meeting; the blue frame shows a connectivity model for interdepartmental consultations, with other members of a single hospital system or in the context of expert consultation practice; the dashed blue line incorporates classic multiple‐header microscope‐type teaching, which can be intra‐ or interdepartmental or interhospital. See text for further explanation.

An overview of activities, pathways and proposed tools is presented in Figure 1.

### Remote Access and Operation for Pathologists

Several pathology departments in the United Kingdom (for example, Leeds and Coventry) and some European centres have established digital pathology‐reporting pipelines. This usually involves conventional dissection and block‐taking from resected surgical specimens or receipt of biopsy specimens, followed by fixation, tissue processing, paraffin‐embedding, sectioning and staining with standard haematoxylin and eosin (and other histochemical) stains or immunohistochemical stains, with the addition of slide‐scanning at the end of this process. Pathologists view the scanned whole‐slide images (WSI), some of which are based on scanners that have already been approved by regulatory agencies for routine reporting,^3^, ^4^ and report them in the conventional way using typical Laboratory Information Management Systems (LIMS). Storage capacities need to be adequately outsourced for those departments that wish to keep a permanent bank of these images; alternatively, departments may decide to only keep the images for a number of years and, if an older slide needs to be revisited, it is then recalled from the physical slide archive and re‐scanned.

Thus, many of the roles and actions of pathologists could be carried out remotely, and the arrangements for this would primarily involve setting up remote access to the WSI viewing system and the LIMS system. Pathologists would also require remote access to clinical databases for cases where clinicopathological correlation involves more detailed interrogation of the clinical and laboratory test result data by the pathologist.

This would also involve remote laboratory interactions, such as computerised laboratory requests (already available in many pathology departments) and remote discussions with histopathology laboratory technical staff via video calling software or, in some cases, telephone calls. Pathology departments would also need to adapt their laboratory etiquette to a new practice, mainly wearing appropriate PPE and/or maintaining a clear social distancing at all times. This may affect the practise of the teaching of description and dissection of surgical specimens from attending pathologists/consultants to trainees/residents. Alternatively, this could be facilitated by the use of video calling software linked to cameras set up to cover the dissection bench, particularly for those trainees who are more experienced.

In general, this accessibility to diagnostic materials and clinical information outside the firewalls of our hospitals would require careful consideration (see Cyber security section below).

The evidence that *in‐silico* reporting represents a significant advantage in the diagnostic process is overwhelming, and its detailed review would be beyond the scope of this article. However, briefly:

1. Digital pathology in leading reference hospitals: the number and quality of biomarker studies and clinical trials in tertiary healthcare are a measure of the quality of healthcare. There is a clear consensus in modern medicine that digital pathology is playing an increasingly important role in these types of studies.^5^, ^6^, ^7^
2. Digital pathology and diagnostic accuracy: the single largest study with more than 3000 samples re‐analysed showed that computer‐based and microscope‐based diagnostic reporting led to similar outcomes.^8^ This will be superseded in 2 years by the results on the ongoing National Institute of Health Research/Health Technology Assessment (NIHR HTA**)** study.^9^
3. Digital pathology and cost‐effectiveness: there are already business case models in place allowing identification of all potential revenue savings associated with the digitalisation of molecular diagnostic services.^10^ These models can serve a single hospital or multiple networks.^11^
4. Digital pathology and laboratory quality: the usual laboratory standards for routine diagnostics are part of the standard accreditation procedures internationally, so a reference framework of quality already exists.^12^
5. Digital pathology and the opportunity for integrating artificial intelligence (AI) into routine pathology practice: the application of AI algorithms will allow a pathology practice of higher quality, lower turnaround time and greater efficiency.^13^
6. Digital pathology and COVID‐19: with the COVID‐19 crisis, many national and international agencies have revisited their criteria to encourage the adoption of digitalisation into routine pathology.^1^, ^2^

### Intradepartmental Activities

Activities described below are based on the assumption that full digitalisation of the diagnostic materials and a degree of digitalisation of the diagnostic services has been achieved by the pathology department.

#### Pathologist‐to‐pathologist intradepartmental consultations

Intradepartmental consultations can be mandatory (subspecialty‐related) or due to diagnostic uncertainty. Regardless of the type, consultations typically occur in two ways; namely, a more informal ‘walk‐down‐the‐corridor’ to share a slide quickly with a colleague to obtain a preliminary impression or a confirmation of a diagnostic impression, or a more formal consultation, where the case requires significant analysis of morphology and immunophenotype. The ideal digital system should allow both. The system should provide a real‐time warning that a new case has come for consultation in either of these two fashions. A real‐time indicator showing that the requested second opinion is available should also be provided. Intradepartmental consultations and/or second opinions should be recorded and attached to the digital documentation of the case, as well as other important aspects of the procedure such as turnaround time. This process may occasionally be more complex; due to the nature of the sample, pathologists may wish to discuss particular aspects of the case by videocall with WSI shared viewing. While digital, real‐time intradepartmental consultations probably began in the context of frozen‐section reporting advice,^14^ the nature of intradepartmental consultations, the reasons why they occur and their unequivocal importance in routine practice has been reviewed recently.^15^ Indeed, a digital intradepartmental service would need to capture all the potential variables in relation to this important (and highly interpersonal) aspect of pathology practice.

#### Group pathologists' intradepartmental discussions

Intradepartmental group discussions typically happen in the context of senior pathologists (and sometimes pathology fellows and senior residents) in subspecialty‐related team meetings. In addition to the considerations on warning of when cases become available for consultation, recording of sometimes complex diagnostic opinions and monitoring of turnaround time, this application would need a very clear model for time and slide management. A chairperson would have the option to provide the ‘active microscope lead’ function to individual members of the discussion group, so that all have the chance of presenting cases as appropriate, including placing emphasis on the morphological, immunophenotypical and other features that they consider key to the diagnosis. Written records of the discussions would also be a prerogative of the chairperson, or these could be delegated to another participant. Final considerations or conclusions resulting from this discussion would be included in the case documentation, with a recommendation for provision of supplementary/addendum reports if necessary.

#### Training of residents

At a time when many have argued for the absolute necessity to incorporate substantial tissue‐ and cell‐based molecular testing into the training portfolio of our residents/trainees,^16^, ^17^ it is important to reflect upon the way in which we conduct the day‐to‐day morphological, immunophenotypical and molecular pathology training. This typically follows two pathways; namely, structured teaching sessions where a series of cases are provided beforehand and discussed ‘around the microscope’, or daily ‘sign‐out sessions’ between a consultant and one or more residents/trainees. Again, the application governing these meetings should attend to all main aspects of the exercise. A classic teaching session with cases chosen beforehand would require a known model for image availability, basic information provision, recording of the trainees' opinions prior to the session and bibliographical references, etc. It would require a system in which the chairperson allows control of the ‘microscope stage’ to pass to individual residents if/when needed. The ‘sign‐out session’ is a much more hierarchical exercise, where a senior pathologist or consultant ‘holds the slides’ in digital form and hence controls the tempo and focus of the session. Thus, for any application that wishes to organise remotely, this process would require full control of ‘microscope stage’ functions, perhaps by use of remote desktop access software or similar, that can be controlled by the consultant or any designated participant, as well as clear connectivity to the LIMS and other hospital clinical information management systems to review draft reports and consult background clinical context and data, allowing the consultant to modify and sign out the case accordingly.

### Intra‐ and Interhospital Activities

Remote intra‐ or interhospital multidisciplinary meetings can take many shapes and forms, from the classic once‐a‐week meetings^18^ to urgent *ad‐hoc* discussions of individual patients. Remote interdepartmental consultations or multidisciplinary meetings (which may include broad multipatient discussions or specific expert consultations between national subject experts) have been recently reviewed.^19^ A total of 33 studies reported the professional, financial and health benefits of multidisciplinary team meetings via telemedicine. Accessibility and availability of expert opinions; improvements in productivity, ergonomics and overtime; significant effectiveness of the use of personal time; cost‐effectiveness; and, long‐term cost reduction were all found to be important. There is overwhelming evidence that remote patient discussion was more efficient before the COVID‐19 crisis, and this is now fully relevant when low‐contact medicine may be preferred in the long term.

The ‘IT management framework’ to run intra‐ or inter‐hospital meetings could be similar and could follow established practices for sharing radiological images currently in use. However, it is the migration of clinical and large histopathological images and metadata outside the confines of the hospital network that makes them a greater challenge, the latter requiring a significant analysis of safety and compliance.

In many ways, these multidisciplinary meetings are highly hierarchical and regulated activities. The best system allowing these to happen in a seamless way would require mechanisms to ensure maximum connectivity to all the participants and teams involved; real‐time access to case images and electronic records; concomitant recording of evidence, discussion and conclusions; monitoring of turnaround time; and, opportunity to ‘step‐in’ and comment on existing images and records while, at the same time, allowing the Chairperson to tightly control the meeting by facilitating interactions and discussions when necessary and appropriate.

Another important routine activity of tissue pathology departments that may be ‘interhospital’ in nature is the training of residents shared by more than one diagnostic team, or delivered to more than one group of residents. This represents a variation of the ‘virtual multiheader microscope’ teaching sessions as described above, but reaching a wider range of teachers and trainees. Indeed, in a discipline in which courses in surgical pathology or histopathology are important for professional training to high standards, reliable tools that are able to facilitate such training for worldwide audiences may become the norm in this new reality.

## Other lessons from the COVID‐19 pandemic

The initial experience with COVID‐19 pathology operations highlight further lessons in the space of healthcare cyber security, clinical sample biobanking and laboratory containment, among others.

### Healthcare Cyber Security

The need for increased interconnectivity calls for a higher availability of diagnostic/clinical images, high‐throughput genomic and molecular pathology information and clinical information, both within and outside the firewalls of hospital information systems. How to allow higher information availability to healthcare providers, including pathologists, while at the same time preserving the individual patient's rights of anonymity and confidentiality, is possibly one of the most difficult conundrums in modern medicine.^20^ Blockchain approaches (the link of clinical records and clinical information using cryptography) have been presented as ways to facilitate privacy‐preserving data access,^21^, ^22^ but we are just beginning to see how correct ISO standards could regulate these processes.^23^ In many aspects, the emerging area of healthcare cyber security (the details of which are outside the scope of this Review) will be at the heart of many of these processes and will dictate what is possible.

### Biobanking

Tissue handling expertise resides in pathology departments, and thus it is the pathologists' duty to coordinate contributions to biobanking that will facilitate research on this and other new pathogens and pathological processes. In situations such as the COVID‐19 pandemic there is a significant need to collect blood samples, material from nasal swabs and other samples, which are usually beyond the scope of traditional tissue‐based banking. However, the previous and the current SARS pandemic is a clear reminder of the importance of autopsy materials to understand the pathogenesis and physiopathology of new diseases.^24^ This includes establishing well‐developed autopsy protocols,^25^, ^26^ as well as resected specimen and biopsy protocols that allow collection of the most important tissue samples, either fresh‐frozen or formalin‐fixed. Indeed, there may be a perception that delays in making these materials widely available may slow the acquisition of basic knowledge of the disease by new research.^27^ Once again, the traditional pathology paradigm of promoting and facilitating the understanding of disease^28^ becomes more important than ever.

These biobanking approaches encourage review of the laboratory containment arrangements. Pathology departments typically receive low‐risk fresh‐frozen samples or any risk sample fixed in formalin. However, the COVID‐19 pandemic has led to some hospitals asking pathology departments to accept high‐risk samples for diagnostics or research sample collection [sputa, bronchoalveolar lavage (BAL) samples and others]. Hence, all pathology departments would be encouraged to develop detailed protocols and training (beyond the scope of this commentary) so that, when necessary, laboratory areas can be remodelled to perform class 3 containment work in modified existing class 2 containment laboratory areas, following existing protocols.^29^

## Conclusions

Telemedicine has been, arguably, one of the areas in the routine practice of medicine that, together with molecular medicine and the application of artificial intelligence, are transforming the way in which medicine is practised. Telemedicine has allowed similar results to face‐to‐face medicine in the management of heart failure,^30^ paediatrics,^31^ acute stroke^32^ or orthopaedic medicine,^33^ to mention a few examples. Telemedicine is used in the fields of nursing^34^ and in the recruitment of patients into clinical trials.^35^ Similarly, telemedicine has been proven to be very effective in the way we teach undergraduate students^36^ in many specific clinical disciplines, such as surgery^37^ or psychiatry.^38^ It is, in many ways, a *modus operandi* that is here to stay.

Some pathology diagnostic services have moved to partial or total digitalisation of their diagnostic material, for purposes ranging from routine diagnosis, training residents and teaching undergraduates to research, or to availability of cases for multidisciplinary team (MDT) purposes and audit. Only a few have embraced digital diagnostic reporting based on viewing digital images. Most are still operating almost exclusively using glass slides and microscopes. The difference between digitalisation of diagnostic materials and digitalisation of the diagnostic service is essential to understand the path that many services still have in front of them for full digitalisation to allow remote digital telepathology practice. In this regard, it is important to understand that some of the technological suggestions provided in this article to allow a full remote service workflow already exist (such as real‐time WSI visualisation), but may need to be revisited when put together with other new tools in the context of a comprehensive solution.

COVID‐19 is a clear reminder that, in addition to many other practical considerations, digital pathology will be a necessary tool in a new reality, where the advantages of pathologists interconnectivity would need to continue in a world of low‐contact professional manners (or professional distancing to complement the social distancing already in place). Our analysis indicates that a series of applications mimicking the best of the pathologist–pathologist, pathologist–clinician and pathologist–trainee interactions are necessary to make the most of the promise of high‐interconnectivity with low contact, a path that may prepare us for a possible new pandemic, but will also make our daily practice easier, richer and diagnostically better.

## Conflicts of interest

The authors have nothing to disclose related to this work.
